# Investigation of Inflammatory Reduction During Extracorporeal Membrane Oxygenation Using a Novel Cytokine Adsorption Column: A Rat Model Study

**DOI:** 10.3390/jcm14051686

**Published:** 2025-03-02

**Authors:** Kota Miki, Hiroaki Fujieda, Yoshiyuki Ueno, Toru Arakane, Yutaka Fujii

**Affiliations:** 1 Medical Engineering Center, Shimane University Hospital, Enya-cho 89-1, Izumo-shi 693-8501, Shimane, Japan; 2 Graduate School, Niigata University of Health and Welfare, Shimamicho1398, Kitaku 950-3198, Niigata, Japan; fujii@nuhw.ac.jp; 3 Toray Industries, Inc., Nihonbashi 2-1-1, Muromachi, Chuo-ku, Tokyo 103-8666, Japan; hiroaki.fujieda.j4@mail.toray (H.F.); yoshiyuki.ueno.p6@mail.toray (Y.U.); toru.arakane.d3@mail.toray (T.A.); 4 Department of Clinical Engineering and Medical Technology, Niigata University of Health and Welfare, Shimamicho1398, Kitaku 950-3198, Niigata, Japan

**Keywords:** extracorporeal membrane oxygenation, rat model, inflammatory, adsorption, polymethyl methacrylate

## Abstract

**Background:** Cardiopulmonary bypass (CPB) and extracorporeal membrane oxygenation (ECMO) are widely used. Previous methods to reduce inflammation have shown inconsistent results. We developed a cytokine adsorption column using polymethyl methacrylate (PMMA) and investigated its anti-inflammatory effects during ECMO. **Materials and Methods:** Male Sprague–Dawley rats were divided into three groups (seven rats in each group): SHAM, ECMO, and ECMO with PMMA (PMMA group). Experiments comprised 180 min of cannulation only in the SHAM group and 60 min of ECMO followed by 120 min of observation in the ECMO and PMMA groups. PMMA adsorption was conducted from 30 min after ECMO initiation to completion in the PMMA group. Blood parameters and cytokines were measured during experiments. Lung tissues were collected after the experiment for evaluation of tissue edema. **Results:** The PMMA group showed significantly lower levels of tumor necrosis factor alpha (TNF-α) and interleukin(IL)-6 compared to the ECMO group at 120 min after completing ECMO. However, there were no significant differences in IL-10 levels between the ECMO group and the PMMA group at the same time points. Lung edema incidence was significantly lower in the PMMA group. **Conclusions:** The PMMA column effectively suppressed systemic inflammatory reactions during ECMO.

## 1. Introduction

Extracorporeal circulation (ECC) support, such as cardiopulmonary bypass (CPB) and extracorporeal membrane oxygenation (ECMO), is widely used in cardiac surgery and acute circulatory failure [1,2]. However, ECC induces various non-physiological conditions and complications, particularly systemic inflammation triggered by blood exposure to foreign surfaces, significantly affecting patient prognosis [3,4,5]. This inflammatory response contributes to endothelial dysfunction, activation of the coagulation cascade, and subsequent organ damage, all of which can complicate ECMO management. As major complications of ECMO treatment, bleeding and infections have gained attention due to their significant impact on clinical outcomes [6,7]. Previous attempts to mitigate inflammation during cardiac surgery using steroids [8,9,10] or miniaturized extracorporeal circuits, which reduce priming volume and blood contact area, have yielded mixed results [11,12]. Additionally, techniques such as modified ultrafiltration during CPB have been investigated to reduce inflammation but have shown inconsistent results in terms of removing inflammatory mediators and improving patient outcomes [13]. Therefore, there is still no standardized approach, and the efficacy of removing inflammatory mediators and improving patient outcomes has been reported to be limited [14,15,16]. In addition to regulating inflammatory responses, infections remain a major complication of ECMO. Prolonged exposure to the extracorporeal circuit increases susceptibility to nosocomial infections, which are associated with increased morbidity and mortality. Furthermore, systemic inflammation can impair immune function, further elevating infection risk. Recent studies suggest that modulating inflammation to support overall immune function, rather than indiscriminately suppressing it, may offer greater clinical benefits [17]. While numerous studies have investigated CPB, it is acknowledged that ECMO, which has advanced significantly in recent years, shares similar ECC-induced inflammatory mechanisms. However, research on inflammation control in ECMO remains limited. Therefore, further development of effective inflammation control technologies is essential [17,18]. However, inflammation is poorly understood and remains an important target for control and intervention [19,20]. Recent reports have also included new inflammatory substances and techniques for controlling inflammation [21,22,23]. We previously attempted to control inflammation using leukocyte removal columns in CPB [24]. However, excessive leukocyte reduction during treatments such as ECMO increased the risk of severe complications from infections. To address this, we focused exclusively on inflammatory cytokines and selected polymethyl methacrylate (PMMA) as the adsorption material. Several studies have reported the use of PMMA to remove inflammatory cytokines, particularly in conditions such as sepsis [25]. The adsorption properties of PMMA have been, and continue to be, studied. It is believed that these properties are determined by the size of the porous membrane, as it adsorbs molecules based on their size, largely independent of the environment. This ensures reliable adsorption as long as the size matches the conditions [23,26]. However, there are few reports of its use during ECMO. Against this background, we developed a cytokine adsorption column using PMMA, designed based on the hypothesis that it selectively adsorbs inflammatory cytokines, such as interleukin (IL)-6 and tumor necrosis factor alpha (TNF-α), while preserving white blood cells (WBCs) and minimizing the removal of anti-inflammatory factors, including high-molecular-weight IL-10. Maintaining a balance between pro-inflammatory and anti-inflammatory cytokines is crucial for preventing excessive immune suppression while still controlling inflammation. In this study, we assessed its efficacy in controlling inflammation and evaluated its impact on the coagulation system using a rat ECMO model. Additionally, we analyzed platelet count variations and coagulation markers to assess the biocompatibility of the adsorption column, as excessive platelet activation and consumption could pose a risk for bleeding complications in ECMO patients.

## 2. Materials and Methods

### 2.1. Animals

Sprague–Dawley rats (male, 14–16 weeks old, 400–450 g) were housed three per cage under a 12 h light–dark cycle with food and water available ad libitum. All animals were provided by CLEA Japan, Inc. (Tokyo, Japan). The animals were brought into the facility and acclimated seven days prior to the experiment.

### 2.2. Ethics Approval

This study protocol was prepared in advance and approved by the Niigata University of Health and Welfare Animal Care and Use Committee (ethical approval no: 22003, date of ethical approval: 23 May 2022). All study protocols were performed in accordance with the National Institutes of Health guidelines for laboratory animal welfare. The experiments were conducted over a period of one year from the date of ethical approval.

### 2.3. Anesthesia, Surgical Preparation, and Extracorporeal Circulation

**Figure 1:**
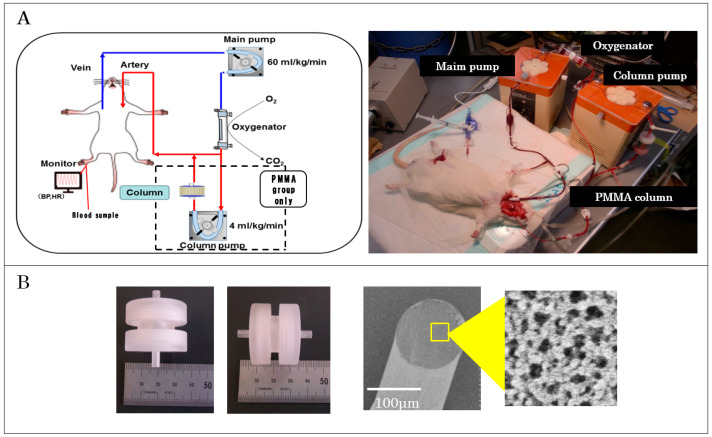
(**A**) Extracorporeal circulation in rat models. (**B**) PMMA columns and structures.

We established a rat model of ECC to efficiently conduct basic research in this field [27]. Previous studies using our rat model have demonstrated the induction of systemic inflammatory reactions by ECMO [28]. The circuit used in the present research comprised a membrane oxygenator (surface area: 0.03 m^2^, polypropylene) and a polyvinyl chloride circuit, with a total priming volume of 15 mL, making this one of the smallest circuits in the world. Anesthesia was induced with isoflurane, followed by orotracheal intubation with a 14 G cannula and ventilation with a respirator (Model SN-480-7; Shinano Seisakusho Co., Tokyo, Japan). Ventilation was volume controlled at a frequency of 70 breaths/min, a tidal volume of 8–10 mL/kg body weight, and inspiration of 100% oxygen. Blood pressure was measured in the femoral artery. The left common carotid artery was then cannulated using polyethylene tubing to deliver blood, and a 16 G cannula was inserted into the jugular vein for blood drainage to establish ECMO at a flow rate of 60 mL/kg/min. During ECMO, arterial partial pressures of oxygen and carbon dioxide were maintained at 300–400 mmHg and 30–40 mmHg, respectively, with a mean arterial pressure above 70 mmHg. We used unfractionated heparin, which is commonly used in clinical practice as an anticoagulant for blood, and monitored activated clotting time as necessary, maintaining it within the range of 200–250 s (Figure 1A). In the case of distress behavior and abnormal vital signs, animals were deeply anesthetized and humanely euthanized promptly.

In the PMMA group, the experiment was conducted by simply attaching the column circuit to the ECMO group circuit, whereas in the ECMO group, no additional circuits were included.

### 2.4. Cytokine Removal Column

We have previously reported on controlling inflammatory reactions associated with CPB and have developed leukocyte removal columns [24]. However, the use of leukocyte removal columns increases susceptibility to infection due to leukocyte depletion. We therefore focused on inflammatory cytokines as the principal mediators of inflammation and developed removal columns using PMMA. PMMA has a porous structure with nano-sized pores and can adsorb various mediators. The structure of the column used in this experiment for rats comprised 4400 fibers filled with PMMA with a diameter of 116 μm (filling rate, 59% by volume). The total contact surface area of the adsorbent was 285 cm^2^, with a filling volume of 1.5 mL (Figure 1B).

### 2.5. Experimental Design

**Figure 2:**
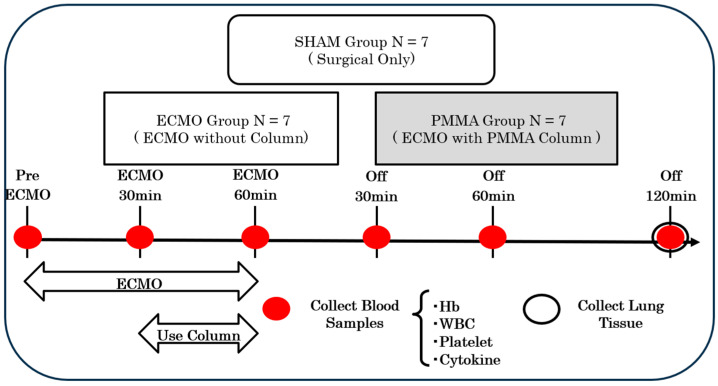
Experiment schedule.

We conducted experiments using a cytokine removal column in a rat model of ECMO. Rats were divided into three groups: a surgery-only group (SHAM group, n = 7), a group that underwent standard ECMO (ECMO group, n = 7), and a group in which the PMMA column was used during ECMO (PMMA, n = 7). The experimental procedure involved 60 min of ECMO followed by a 120 min observation period after the completion of ECMO. In the group using the PMMA column, blood was perfused through the column parallel to the ECMO-sending circuit at a rate of 4 mL/kg/min (equivalent to 200–300 mL/min in humans, assumed for experimental termination) from 30 to 60 min after initiation of ECMO until the end of the experiment. A schematic diagram and photograph of the rat PMMA group are shown in Figure 1A. In the PMMA group, the column was filled 30 min after ECMO initiation. This staggered approach was adopted to prevent excessive dilution from occurring all at once and to mitigate its impact. Arterial blood was collected from the arterial line before the initiation of ECMO and every 30 min thereafter until the end of the experiment, to allow measurement of WBC count, platelet count, and levels of the cytokine TNF-α, IL-6, and IL-10. In addition, lung tissue was collected at the end of the experiment, and the wet-to-dry ratio was measured to evaluate the degree of lung tissue edema (Figure 2).

This figure illustrates the experimental design and time course. ECMO was initiated at 0 min and continued for 60 min in both ECMO and PMMA groups. In the PMMA group, the adsorption column was used during ECMO. After ECMO cessation (off), animals were observed for an additional 120 min. Blood samples were collected at multiple time points (pre-EMCO, after ECMO initiation 30 and 60 min, and post-ECMO 30 and 60 min, 120 min) to analyze hemoglobin (Hb), WBC count, platelet count, and cytokine levels. Lung tissue was collected at the end of the experiment (120 min post-ECMO cessation).

Blood dilution was corrected by converting hemoglobin (Hb) levels to hematocrit (Ht) values and calculating the plasma ratio before and after dilution (1-Ht/100) to obtain the adjusted values. All animals were humanely euthanized at the end of the experiment by potassium chloride injection. The superior third of the lung was used to calculate the wet-to-dry ratio at the end of the experiment. The superior part of left lung was harvested and used for the calculation of W/D ratio. The lung block was standardized to a size of approximately 27 mm^3^ with dimensions of 3 mm × 3 mm × 3 mm. The lung block was weighed before and after desiccation for 72 h in a dry oven at 70 °C.

### 2.6. Statistics

In this study, the sample size was determined with “wet to dry” as the primary endpoint. A power analysis was conducted with the goal of achieving a statistical power of 0.80 and an alpha level of 0.05. This analysis indicated that at least 6 subjects per group would be required to detect significant differences for the primary endpoint. The data used had similar distribution shapes and were independent continuous data, but normal distribution could not be confirmed for some of the data. Therefore, ANOVA tests were conducted for blood pressure, heart rate, and hemoglobin, followed by post-hoc analysis using the Tukey test. For other items, the Kruskal–Wallis test was used. Post-hoc comparisons were performed using the Steel–Dwass multiple comparison test to identify specific group differences. Values of *p* < 0.05 were considered to indicate a statistically significant difference. Data for body weight mean blood pressure, heart rate, and hemoglobin were expressed as mean ± standard deviation (SD), and other items were expressed as median (first quartile—third quartile). All statistical analyses were performed using EZR (Saitama Medical Center, Jichi Medical University, Saitama, Japan), a graphical user interface for R version 1.64 (The R Foundation for Statistical Computing, Vienna, Austria) [29].

## 3. Results

**Figure 3:**
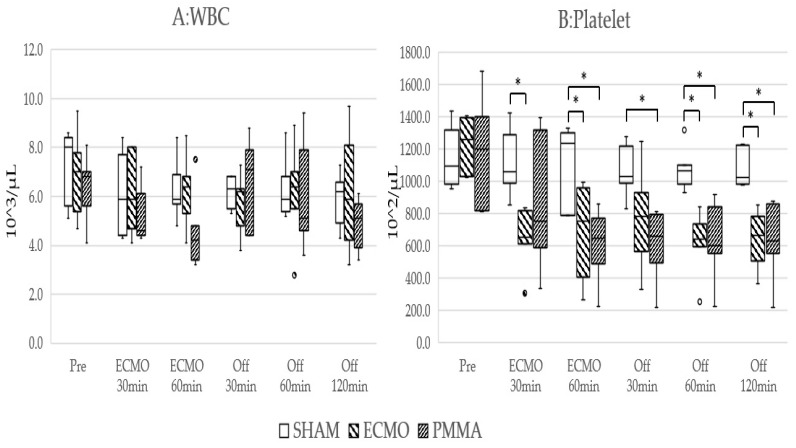
Changes in blood counts during the experiment. (**A**) WBC count. (**B**) Platelets count. * *p* < 0.05.

**Figure 4:**
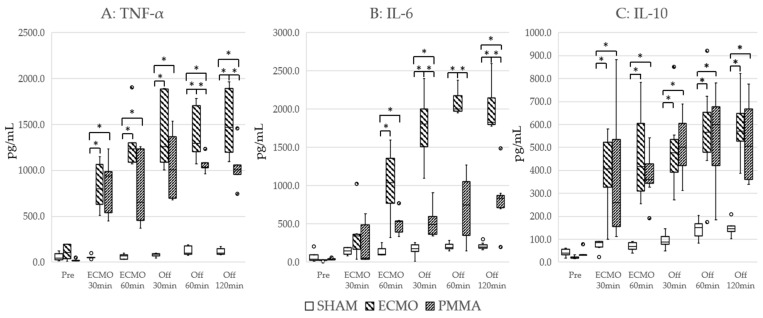
Changes in cytokine levels during the experiment. (**A**) TNF-α. (**B**) IL-6. (**C**) IL-10. * *p* < 0.05.

**Figure 5:**
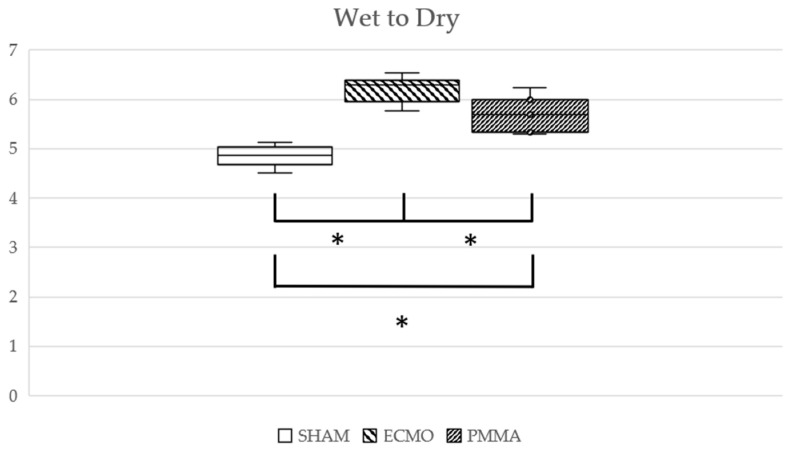
Lung wet-to-dry weight ratio. * *p* < 0.05.

No animals were excluded due to hypotension or abnormal vital signs from the start of the experiment. Hemodynamics during the experiment are shown in Table 1. WBC and platelet counts are shown in Figure 3, cytokine levels in Figure 4, and lung tissue wet-to-dry weight ratio in Figure 5. The values of white blood cells and platelets, adjusted for the effects of blood dilution, are presented in the Supplemental Figure S1. The significant differences of each parameter are shown in the Supplemental Table S1. The body weights of the rats were as follows: SHAM group 437.4 ± 19.1 g, ECMO group 444.9 ± 15.9 g, and PMMA group 434.9 ± 29.4 g. There were no significant differences between the groups. At the start of each experiment, no significant differences among groups were apparent for any parameter.

After 30 min of ECMO, WBC counts decreased by 12.8% in the ECMO group and 16.6% in the PMMA group. At 60 min after ECMO initiation, compared to the after 30 min into ECMO, WBC counts increased by 2.8% in the ECMO group but decreased further by 16.7% in the PMMA group. Platelet counts decreased by 45.5% and 27.9%, respectively, at 30 min, and at 60 min they increased by 2.3% in the ECMO group while further decreasing by 27.5% in the PMMA group. Levels of IL-6, IL-10, and TNF-α increased significantly in both the ECMO and PMMA groups. Hemoglobin levels at 30 min into ECMO did not differ significantly between the ECMO and PMMA groups. Calculated blood dilution rates from hemoglobin levels at 60 min into ECMO (after column use) were 31.8% in the ECMO group and 36.1% in the PMMA group. In the PMMA group, WBC counts decreased from 4600 (4450–6100)/μL to 4200 (3500–4550)/μL and platelet counts decreased from 756 (651–732) × 10^2^/μL to 647 (545–732) × 10^2^/μL after column usage. However, no significant differences in WBC and platelet counts were observed between the PMMA and ECMO groups at any time point. In addition, WBC count remained at 5100 (4300–5550)/μL even 120 min after ECMO cessation. Furthermore, levels of the inflammatory cytokines IL-6 and TNF-α had a lower rate of increase from ECMO start to 60 min in the PMMA group compared to the ECMO group. The increase in IL-6 at 30 min after ECMO cessation was significantly lower in the PMMA group compared to the ECMO group. TNF-α levels were also lower in the PMMA group at 60 min after ECMO cessation and showed a decreasing trend at 120 min. Furthermore, in the TNF-α, both the ECMO and PMMA groups exhibited similar increases 30 min after ECMO initiation. However, from 60 min after ECMO initiation (post-column use) to 30 min after ECMO cessation, TNF-α levels in the PMMA group remained lower, although the difference was not statistically significant. At 60 min post-ECMO, TNF-α levels were significantly lower in the PMMA group and continued to decline at 120 min. For IL-6, levels showed a decreasing trend after column use and were significantly lower in the PMMA group compared to the ECMO group from the end of ECMO cessation. In contrast, the anti-inflammatory cytokine IL-10 was not significantly affected by PMMA adsorption. Although IL-10 levels showed a slight tendency to be lower after column use and at 120 min post-ECMO, they remained comparable to those in the ECMO group without statistical significance. At the end of the experiment, the lung wet-to-dry ratio was 4.87 (4.75–4.97) in the SHAM group, 6.29 (6.05–6.34) in the ECMO group, and 5.69 (5.51–5.90) in the PMMA group, with significant differences observed among the groups.

## 4. Discussion

Hemodynamic parameters were maintained within the target range throughout the experiment. However, at 60 min after ECMO initiation, blood pressure in the PMMA group was lower compared to that in the SHAM group, suggesting that the effect of blood dilution caused by the PMMA column exceeded the circulatory support provided by ECMO. In the PMMA group, the WBC count decreased by 17% after column usage; however, when adjusted based on the provided Hb data, the decrease was only 0.5%. Additionally, no significant differences in WBC counts were observed between the PMMA and ECMO groups 60 min after ECMO completion. These findings suggest that the impact of the PMMA column on WBCs is minimal.

Moreover, the increase in inflammatory cytokines (TNF-α and IL-6) was suppressed, suggesting cytokine adsorption independent of WBC removal. These differences persisted even 120 min after ECMO cessation. In contrast, in the PMMA group, IL-10 levels increased similarly to those in the ECMO group, indicating that IL-10 was not removed, whereas the other cytokines were effectively adsorbed. Notably, adsorption depends on the size of the target substances. Therefore, IL-6 and TNF-α, which have molecular weights of approximately 20 kDa, are likely to be adsorbed, whereas IL-10, which forms a dimer and reaches a molecular weight of around 40 kDa, is less likely to be adsorbed. This suggests that the column used in this study maintained an immune balance by suppressing pro-inflammatory mechanisms while preserving immunosuppressive functions. The reduction in lung wet-to-dry ratio in the PMMA group suggests that the PMMA column controlled inflammation during ECMO, directly maintaining normal vascular permeability and contributing to the alleviation of pulmonary edema.

Furthermore, no significant differences in platelet reduction were seen between the PMMA and ECMO groups, indicating sufficient biocompatibility. However, despite the absence of significant differences, a 2.2% decrease in platelet count was noted in the PMMA group at 60 min after ECMO initiation, even after accounting for dilution effects. In this study, the PMMA column was used for a short duration of 30 min; therefore, further investigation is needed to assess its impact on platelets over longer periods. Research on the biocompatibility of PMMA is ongoing, with advancements in technologies aimed at minimizing platelet adsorption, which are expected to further improve its biocompatibility [30]. Systemic inflammatory response syndrome (SIRS) is associated with high cytokine levels during ECC and is known to significantly affect patient outcomes after ECC [3,4,5,6]. Several techniques, including reducing the priming volume and blood contact area during CPB [10,11], and MUF [12,13,14], have been explored as measures to mitigate inflammation. However, reports have suggested mixed outcomes regarding the effectiveness of these techniques. Blood adsorption has been used for immune regulation, with PMMA widely used in continuous renal replacement therapy for acute renal failure and sepsis [23,25] and showing effectiveness in adsorbing inflammatory substances.

In the present study, the newly developed PMMA column for ECMO demonstrated the potential to adsorb representative inflammatory cytokines and effectively suppress systemic inflammatory reactions and organ damage induced by ECMO. Substances that potentially play significant roles in SIRS are under continuous investigation, and not all aspects have been fully elucidated. However, the PMMA column used here has demonstrated potential to adsorb numerous inflammatory substances, including those that remain incompletely understood [22,23,31,32]. This validation was conducted over a short period; however, ECMO management in clinical practice is typically prolonged. ECMO is highly invasive, and ensuring stable management during the early phase without causing treatment setbacks is a critical challenge. Additionally, the ELSO guidelines highlight infection and bleeding as major risks associated with ECMO initiation. Regarding biocompatibility, the developed column utilizes clinically approved materials, ensuring a certain level of safety. This study demonstrated minimal impact on platelets, which are essential for hemostasis during ECMO, and little effect on WBCs, suggesting that the column does not induce excessive immunosuppression. These findings suggest that the column may exert specific and beneficial effects in ECMO therapy. Moving forward, further exploration of the inflammatory substances adsorbed by the PMMA column, validation of its capacity to remove inflammatory substances generated during ECC-induced SIRS, and precise measurement of the upper limit of cytokine adsorption capacity will be essential.

## 5. Conclusions

Using a rat ECMO model, this study demonstrated that cytokine adsorption by the PMMA column effectively controlled inflammatory reactions, as evidenced by pathological improvements in the lung based on the wet-to-dry ratio. Additionally, the findings suggest that, for short durations, the PMMA column can help regulate inflammation while minimizing its impact on white blood cells and platelets. However, further studies are needed to evaluate its adsorption capacity and long-term safety.

## Figures and Tables

**Table 1:** Hemodynamics during the experiment.

	Group	Pre	ECMO 30 min	ECMO 60 min	OFF 30 min	OFF 60 min	OFF 120 min
MAP [mmHg]	SHAM	104 ± 4	93 ± 6	98 ± 6	94 ± 5	100 ± 5 *	97 ± 4
	ECMO	102 ± 4	88 ± 3	84 ± 2	83 ± 4	83 ± 4	80 ± 5
	PMMA	98 ± 3	76 ± 3 *	74 ± 4	75 ± 5	75 ± 3	72 ± 3
HR [bpm]	SHAM	385 ± 16	399 ± 12	366 ± 11	385 ± 11	378 ± 6	386 ± 12
	ECMO	387 ± 5	384 ± 9	371 ± 5	364 ± 6	363 ± 11	357 ± 9
	PMMA	370 ± 6	387 ± 8	362 ± 8	353 ± 8	351 ± 11	355 ± 11
Hb [g/dL]	SHAM	14.1 ± 0.5	13.7 ± 0.5	13.9 ± 0.5 *	13.4 ± 0.3 *	13.0 ± 0.6	12.8 ± 0.6 *
	ECMO	13.6 ± 0.4	10.7 ± 0.8	9.3 ± 0.3	11.0 ± 0.5	11.0 ± 0.8	10.0 ± 0.3
	PMMA	13.9 ± 0.4	9.4 ± 0.4	8.7 ± 0.8	9.4 ± 0.5	10.1 ± 0.9	9.6 ± 0.7

Data are expressed as mean ± SD. MAP: mean arterial pressure. * *p* < 0.05 compared to ECMO group.

## Data Availability

All raw data are available upon reasonable request.

## Supplementary Materials

The following supporting information can be downloaded at: https://www.mdpi.com/article/10.3390/jcm14051686/s1.
