# Supplementary figures and images for: Investigation of Inflammatory Reduction During Extracorporeal Membrane Oxygenation Using a Novel Cytokine Adsorption Column: A Rat Model Study
Source: J Clin Med. 2025 Mar 2;14(5):1686. doi: 10.3390/jcm14051686 (PMC11900994; doi:10.3390/jcm14051686)

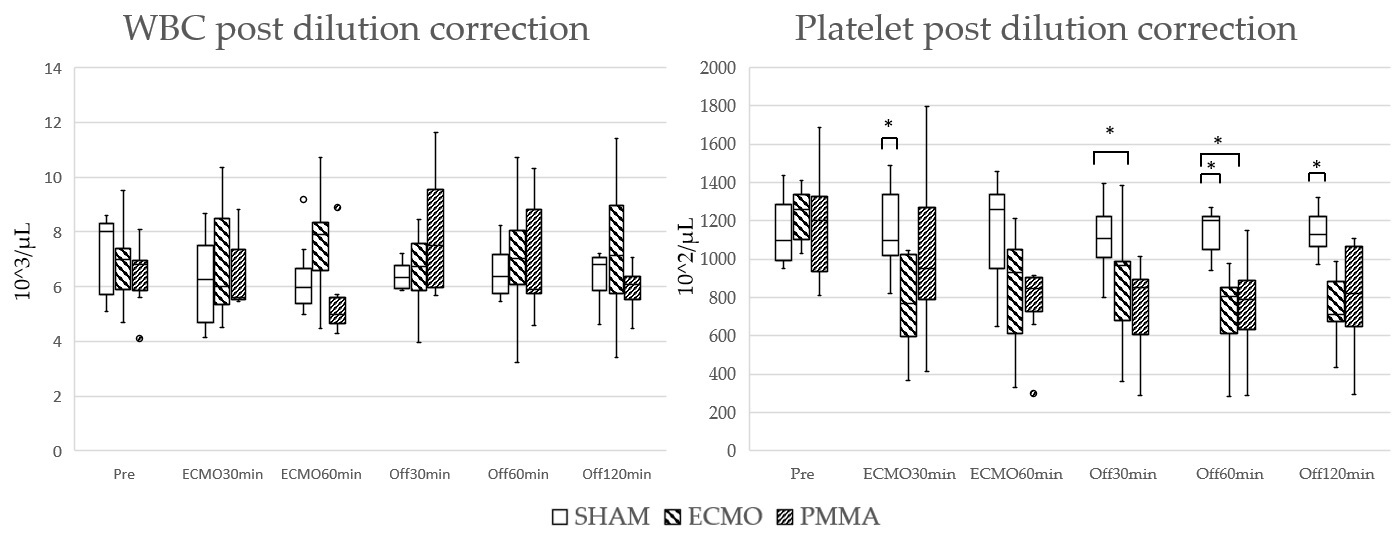

Supplement: Supplementary file 1 [file jcm-14-01686-s001.zip › supplemental Figure S1.jpg]

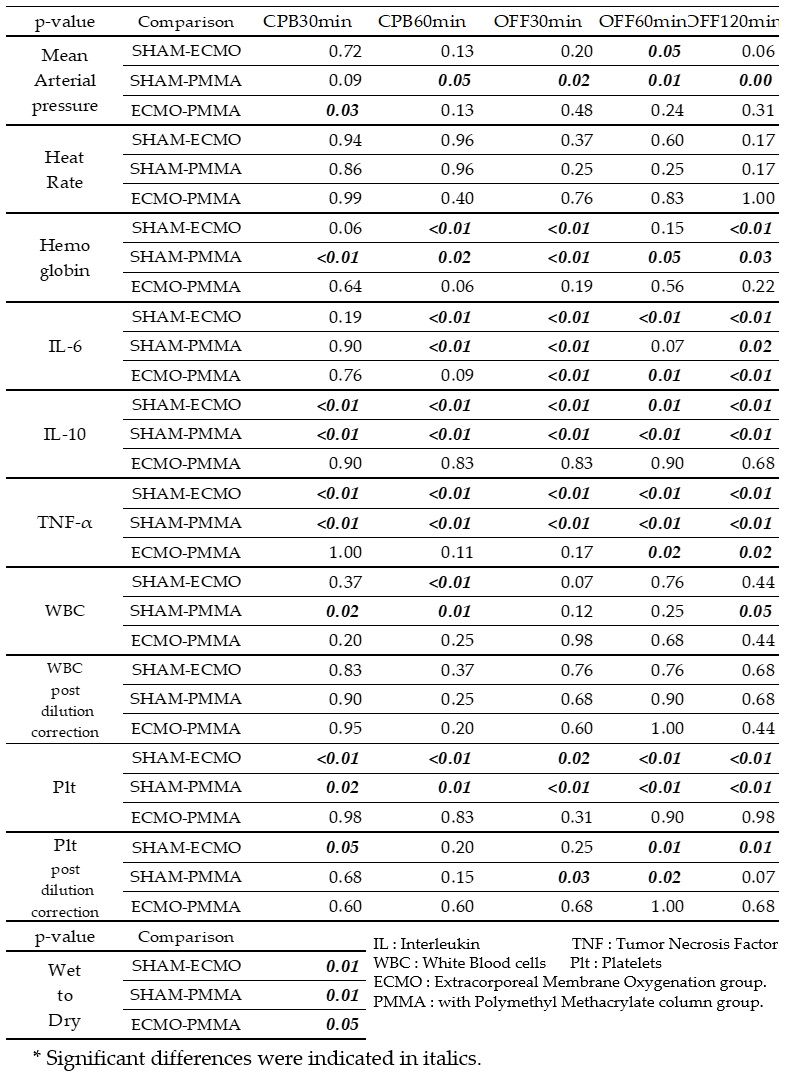

Supplement: Supplementary file 1 [file jcm-14-01686-s001.zip › supplemental Table S1.jpg]
